# Coupling quantitative systems pharmacology modelling to machine learning and artificial intelligence for drug development: its *pAIns* and *gAIns*

**DOI:** 10.3389/fsysb.2024.1380685

**Published:** 2024-07-12

**Authors:** Núria Folguera-Blasco, Florencia A. T. Boshier, Aydar Uatay, Cesar Pichardo-Almarza, Massimo Lai, Jacopo Biasetti, Richard Dearden, Megan Gibbs, Holly Kimko

**Affiliations:** ^1^ Systems Medicine, Clinical Pharmacology and Safety Sciences, R and D BioPharmaceuticals, AstraZeneca, Cambridge, United Kingdom; ^2^ Systems Medicine, Clinical Pharmacology and Safety Sciences, R and D BioPharmaceuticals, AstraZeneca, Gothenburg, Sweden; ^3^ Machine Learning and AI, Clinical Pharmacology and Safety Sciences, R and D BioPharmaceuticals, AstraZeneca, Cambridge, United Kingdom; ^4^ Clinical Pharmacology and Safety Sciences, R and D BioPharmaceuticals, AstraZeneca, Waltham, MA, United States; ^5^ Systems Medicine, Clinical Pharmacology and Safety Sciences, R and D BioPharmaceuticals, AstraZeneca, Gaithersburg, MD, United States

**Keywords:** quantitative systems pharmacology (QSP), machine learning (ML), artificial intelligence (AI), drug development, real world data (RWD), mathematical modelling, data heterogeneity, precision medicine

## Abstract

Quantitative Systems Pharmacology (QSP) has become a powerful tool in the drug development landscape. To facilitate its continued implementation and to further enhance its applicability, a symbiotic approach in which QSP is combined with artificial intelligence (AI) and machine learning (ML) seems key. This manuscript presents four case examples where the application of a symbiotic approach could unlock new insights from multidimensional data, including real-world data, potentially leading to breakthroughs in drug development. Besides the remarkable benefits (*gAIns*) that the symbiosis can offer, it does also carry potential challenges (*pAIns*) such as how to assess and quantify uncertainty, bias and error. Hence, to ensure a successful implementation, arising *pAIns* need to be acknowledged and carefully addressed. Successful implementation of the symbiotic QSP and ML/AI approach has the potential to serve as a catalyst, paving the way for a paradigm shift in drug development.

## Introduction

Mathematical modelling of biological processes has traditionally been performed by integrating observations from in-house experiments and/or published literature; understanding the physiological/pathological/pharmacological mechanisms of action; and, under simplifying assumptions, translating such processes into equations that can represent the observed dynamics. Due to their mechanistic underpinnings, these models do not necessitate extensive datasets for development: they leverage scientific knowledge of the underlying processes, as opposed to being data-driven. Moreover, they can be used to fill in a gap when data is lacking or scarce, as they provide a means to test hypotheses in a less costly manner. Once built, model exploration can predict how biological systems will behave under distinct conditions, characterising mechanisms not observed experimentally. The pharmaceutical industry has been using mathematical modelling to support many phases of drug development, and nowadays, Quantitative Systems Pharmacology (QSP) modelling has become a popular approach in multiple facets: helping to make go/no-go decisions for drug development investment, finding optimal doses and dosing schedules for monotherapies and/or combination therapies, providing valuable input in regulatory decisions, and so forth (Azer et al., 2021; Jane et al., 2021; Verma et al., 2023).

QSP model development requires a lot of time and effort commitment as it involves extensive literature curation and a deep understanding of the underlying biology/pharmacology, normally spanning multiple scales, which also challenges data integration during its validation. However, novel approaches based on machine learning (ML) and artificial intelligence (AI) are expected to facilitate such a process. ML/AI-based models, normally used for rich/large datasets, apply relevant algorithms to learn/identify patterns in the data. By exhaustively exploring such comprehensive datasets**,** even in the absence of *a priori* mechanistic knowledge—a requisite for QSP approaches—ML/AI may narrow down plausible mechanistic options which can then be further validated by a QSP model. This process holds significant potential for uncovering the mechanisms behind unreported phenomena, such as emerging side effects associated with novel therapies.

Therefore, current efforts are focused on creating a mutualistic symbiosis between the QSP and ML/AI fields [see (Procopio et al., 2023) for some examples], aiming to achieve improved overall results, more informative than either approach alone. Since QSP and ML/AI have different data requirements and data-integration capacities, their symbiotic relationship can consist in: (i) consecutive application, where one approach tackles a specific stage, and the (partial) results are used by the other methodology, or (ii) simultaneous application, where both approaches work together on the same data. In the consecutive case, a ML/AI approach followed by QSP could yield mechanism discovery when mechanistic knowledge is lacking, whilst using a QSP model first could help to improve performance accuracy of ML/AI algorithms, by generating training data. When applying QSP and ML/AI simultaneously, researchers can leverage the strengths of each method to integrate diverse data sources. This is particularly beneficial because a single approach might not be able to handle all the data types (e.g., imaging data, quantitative and/or qualitative data), but the combined approach can utilise the full potential of this rich data landscape.

**FIGURE 1:**
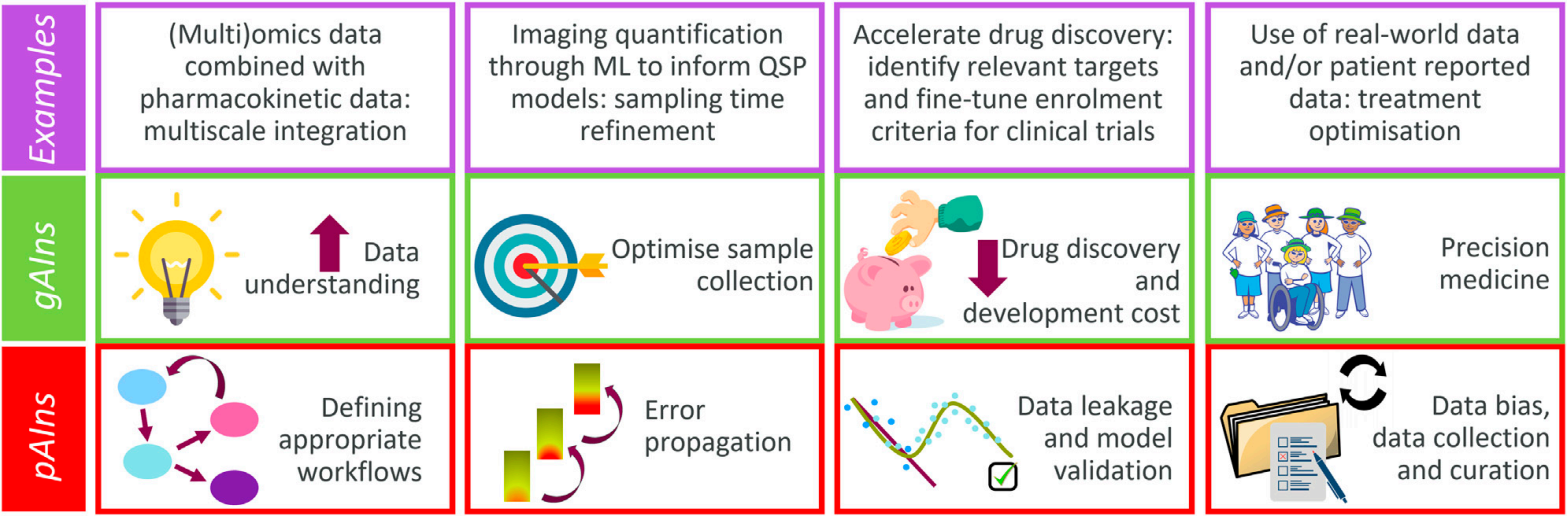
Major *gAIns* and *pAIns* arising from the QSP and ML/AI symbiotic implementation (contextualised with examples discussed).

This paper shows current cases being explored at AstraZeneca that highlight the prospective benefits (*gAIns*) of combined QSP-ML/AI approaches, followed by a discussion around some of the concerns (*pAIns*) that this symbiosis may bring (Figure 1). At the current early stage of this collaboration, the balance between *gAIns* and *pAIns* seems to be tilted towards the former. However, acknowledging and addressing the *pAIns* will ensure that the ML/AI and QSP symbiosis becomes truly a beneficial one.

## *gAIns* of combined QSP-ML/AI approaches

**FIGURE 2:**
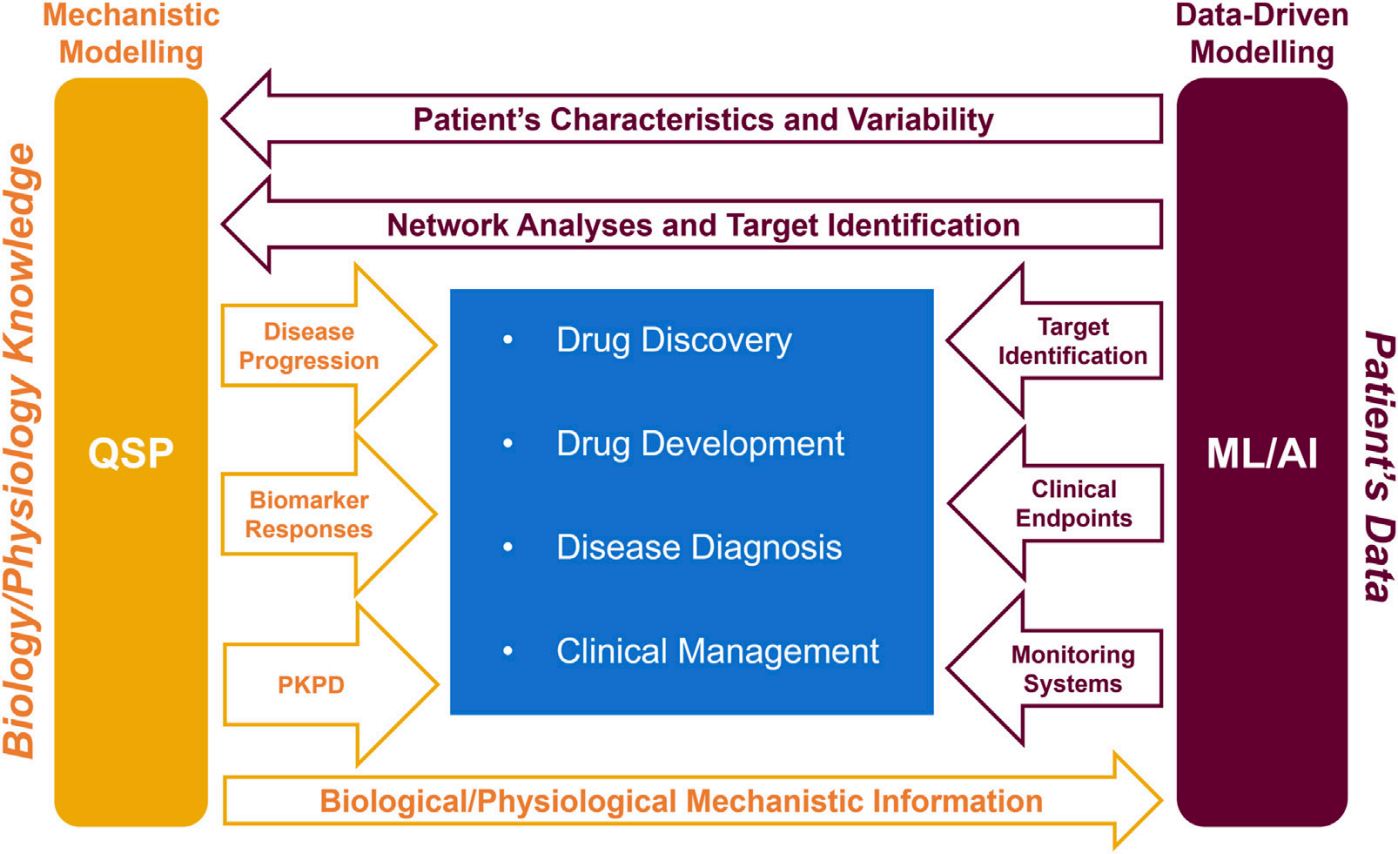
Schematic representation of how the symbiotic QSP and ML/AI approach can help the pharmacological healthcare continuum (drug discovery, drug development, disease diagnosis, and clinical management). PKPD, pharmacokinetics and pharmacodynamics.

This section describes 4 case examples which highlight how the QSP and ML/AI symbiotic approach presents an advantage *versus* using just one single methodology. The scenarios described have been selected to span a wide spectrum in the drug research landscape, ranging from implications at the cellular level to clinical stages (Figure 2). Therefore, the symbiotic approach may have significance both for patients and for pharmaceutical industries, as it may help designing better disease treatments, and ultimately, improve patients’ quality of life.

### Cellular level: multi-omics data integration

Omics data capture multiple facets of cell signalling, including gene transcription (transcriptomics), translation (proteomics), and metabolism (metabolomics). Technological advancements have led to a remarkable accumulation of these data, which has spurred the development of ML methods for processing each data layer (Li and Li, 2018; Liebal et al., 2020; Neely et al., 2023). Despite the advances in ML and QSP methods, it is challenging to leverage omics data in QSP modelling (Zhang et al., 2022; Uatay et al., 2023). These challenges include both technological (e.g., scarcity of information on kinetic aspects of cell signalling) and methodological (e.g., how to interpret multi-layered data) limitations.

Combined ML and QSP approaches often involve dimensionality reduction (e.g., principal component analysis) and identification of enriched genes (or proteins, metabolites), which are overlayed on pre-built signalling networks or used to construct a model of the relevant pathways (Zhang et al., 2022). Some of the limitations of this approach are due to the static nature of the data (only a snapshot of cellular signalling is captured) and/or limited (if not absent) utilisation of complementary information, which integrated analysis of several layers of omics data can provide. Since QSP describes dynamics across multiple layers of cell signalling simultaneously (transcription, translation, and metabolism are interlinked processes), advances in ML methods capable of extracting real time-course information about these processes [as opposed to latent-time approaches (Bergen et al., 2021)] may provide a new impetus for the development of the QSP field. While latent-time approaches can infer kinetics of gene expression on a (relative) timescale determined by a given (single cell RNA-seq) dataset, such data is not directly compatible with pharmacokinetics data—a critical input for QSP. Therefore, inference of cell-signalling dynamics on an absolute timescale may facilitate integration of molecular scale processes into a QSP model.

In the last years, numerous methods, including ML-based ones, have been developed to integrate multiple omics layers to generate mechanistic hypotheses about the overall state of cell signalling (Garrido-Rodriguez et al., 2022). However, the development of such ML methods for multi-omics integration is challenging due to limited data availability and incomplete knowledge about “ground truth.” Nonetheless, incorporation of prior knowledge can facilitate reconstruction of the (perturbed) signalling network and can potentially enhance the predictive power (Dugourd et al., 2021). As QSP integrates prior knowledge with contextual data (e.g., disease, efficacy, and safety) in a quantitative and mechanistic manner, which can then be used to generate synthetic but realistic training data for ML models, there is a synergistic potential between QSP and ML-based methods for multi-omics analysis. Such synergy may become especially useful in ML models such as deep neural networks (e.g., via a framework provided by physics/biology-informed neural networks), where incorporating prior knowledge is particularly challenging.

Recently, an ML approach was developed to model the dynamics of gene regulatory networks during haematopoiesis by leveraging technological advancements in single-cell RNA-seq (scRNA-seq) (Qiu et al., 2022). Such a work showcased how technological (ability to resolve temporal aspects of transcription) and methodological (ML/modelling) advancements facilitated overcoming one of the limitations of scRNA-seq—i.e., lack of temporal information. Combining this methodology with a QSP model to characterise the effect of drug- or disease-induced perturbation could be an excellent example of ML/AI-powered *gAIn* for QSP because the ML approach is unbiased, comprehensive, and amenable to mechanistic modelling via traditional QSP tools such as ODEs.

Advances in single-cell omics technologies also include inferences of spatially resolved information (Alexandrov et al., 2023). Development of ML/AI methods to process spatiotemporal aspects of cell signalling, as well as different omics layers, has the potential to enhance QSP predictions by removing key limitations (e.g., snapshot of a regulatory network and/or a single signalling layer) of omics-powered modelling and/or by improving accuracy via an additional layer of validation of the predictions. One such method might involve combining AI-powered cell segmentation on tissue slices with mass spectrometry imaging, which may ease single-cell level omics analysis, and facilitate its utilisation in QSP models.

### Tissue level: quantification of imaging data for QSP modelling

Biological samples are extremely valuable in R&D departments, as they provide a means to better understand disease and assess therapy efficacy or safety. However, those samples tend to be scarce, might involve invasive treatments for patients and incur high costs. Therefore, to gain the most insights, it is needed to optimise both what information to be extracted from those samples and the timing of their collection.

Complex image segmentation tasks for the analysis of pathology images rely on advanced deep learning algorithms, where deep convolutional neural networks (CNNs) excel at learning and extracting intricate cellular patterns. The usefulness of processing pathology images by CNNs lies in its ability to consistently, efficiently, and precisely distinguish among different cell types under a variety of imaging conditions, such as staining density and illumination variability (Hosny et al., 2018). Additionally, the segmented images are perfectly suited for the derivation of quantitative measurements essential for downstream QSP models. Such quantification could be done manually, but AI has been proven successful in outperforming hand-crafted techniques. Furthermore, such a pipeline can generate much larger volumes of data than manual approaches, which allows AI to optimise QSP model parameters to best match the imaging data, as well as permit QSP models to be validated on very large imaging datasets.

A clear application of such a tool is in the development of a stem cell therapy for heart failure, where human ventricular progenitor cells are injected into infarcted myocardial tissue with the aim of regenerating the damaged muscle (Timmer and van Rooij, 2002; Foo et al., 2018; Poch et al., 2022). A key step in the development of this therapy is the reliable quantification of ventricular progenitor cell engraftment, i.e., how many cells are retained in the injured myocardium and connect with the host tissue. Moreover, given the limitation in the quantity of longitudinal data points that can be collected, a QSP model of host-graft tissue evolution is needed to predict efficacy and safety of the therapy.

Thus, to support therapy development, an imaging-driven systems model of host-graft tissue evolution can be developed. First, slices of cardiac tissue from preclinical studies are stained and digitised. The images then need to be labelled at the cellular level by an expert to allow cell segmentation and identification. Such labels should be used to train a ML model to detect host and graft cells based on their shape and stain colour. Since individual cell labelling is very time consuming, methods to fine-tune existing models [e.g., able to identify more cell types or capable of using the labels of the whole image for training (Mokhtari et al., 2023)] hold great promise. The use of such a ML model on newly generated pathology images provides a means to reliably obtain data on tissue cellular composition and its spatial characterisation (e.g., tissue size, shape, relative position). Such data is then used for subsequent QSP modelling of tissue evolution.

The described AI-based pipeline for data extraction from cardiac pathology images shows the relevance of obtaining quantifiable measurements, which can then be integrated in QSP approaches, to understand complex cardiac behaviours. More generally, this case example highlights the key role that an integrated approach may play in the development of predictive models for cell therapy strategies, as it may provide additional insights which can inform optimal times for future sample collection.

### Clinical level: modelling heterogeneous data types

Continuous progresses in ML/AI tools are easing the use of real-world data, which holds promising potential to unveil relevant information for treatment discovery and development. Despite such technical advances, the use of clinical data is not straightforward. Clinical data is highly heterogeneous as it encompasses, among other data types, patient’s clinical records (e.g., demographics, family health history, previous diagnoses, medications, patient-reported outcome measures) and biomarker/imaging data (e.g., genetic sequences, lab chemistry results, X-rays, CT scans). The difficulty in integrating such data is well exemplified when trying to connect qualitative clinical endpoints (e.g., symptoms, functional status, or disease severity) to quantitative measurements (e.g., blood cell count time-series). Current efforts are focused on trying to draw a link between such data, aiming to obtain some (mechanistic) understanding of clinical response to treatment.

For complex biological therapies such as those involving T-cell engagers, predicting potential side effects, like cytokine release syndrome (CRS), remains a challenge. While QSP models can effectively predict continuous quantitative biomarkers (e.g., cytokines), their limited understanding of CRS mechanisms often hinders their ability to directly predict such a critical outcome (presence/absence of CRS and its severity grade). This is where a symbiotic QSP and ML/AI approach may provide a reasonable bridging strategy; by harnessing the power of real-world data, ML/AI approaches can fill the knowledge gap in CRS prediction by revealing the relationship between patient features and CRS incidence. A QSP model, although unable to predict CRS by itself, can provide quantitative data, such as predicted biomarker levels based on estimated T-cell engager trimer exposure (e.g., longitudinal IL-6 profiles) (Chen et al., 2019; Hosseini et al., 2020; Weddell, 2023). Quantitative predictions from the QSP model can then be fed into a ML/AI-driven risk prediction model built from real-world patient clinical characteristics and CRS observations, to establish a predictive model for CRS risk. This integrated approach holds significant promise for improving patient safety and optimising treatment regimens (Irons et al., 2023).

### Bench-to-bed translation: from drug development to precision medicine

To progress towards better disease understanding and treatment, untangling disease intricacies is a requisite, for which QSP modelling may become helpful, as it allows to simulate different hypothetical scenarios. The increased understanding can help in identifying novel therapeutic mechanisms/targets. Once identified, the targets can then be leveraged by ML/AI algorithms to design effective diagnostics, optimise clinical trials, and personalise treatment regimens. Therefore, a symbiotic QSP and ML/AI approach may offer a powerful engine for personalised healthcare, spanning from drug development to precision medicine implementation. To exemplify this, an outline on how such a combined approach could comprehensively help in the research and treatment of Alzheimer’s disease (AD) is presented (see Figure 2 for a graphical representation).

#### Drug discovery and development

QSP and AI approaches have both been used for AD drug development. For example, an AD progression mechanistic model to capture the complex interplay between amyloid aggregation, pharmacokinetics, and biomarker dynamics has been developed (Geerts et al., 2023). The QSP model supported clinical trial design, helping to define optimal schedules based on understanding biomarker response variability. On the ML/AI side, AI-powered algorithms have been used to analyse patient data to both identify the most promising drug targets and predict how patients will respond to different treatments (Myszczynska et al., 2020), which may facilitate decisions on subject enrolment criteria to improve the probability of success in clinical studies. Therefore, the QSP and AI/ML symbiotic approach can help to understand and compare the efficacy/safety of drug candidates addressing different targets by analysing the dynamics of relevant biomarkers and incorporating the inherent heterogeneity observed in AD patients.

#### Disease diagnosis

AI-powered algorithms can analyse brain imaging data to detect AD earlier and more accurately than traditional methods can, reaching 99% accuracy levels (Odusami et al., 2021). They can also be used to understand face recognition deficits (Singh and Ramanathan, 2023). Face recognition datasets can be used to train AI models, whose recognition accuracy decreases as they are perturbed to emulate brain tissue dysfunction. Such results indicate that, despite neural networks not being an exact representation of biological neuronal activity, an AI framework could potentially mimic the loss of cognitive function in AD patients. However, such AI-based models did not include time as an explicit predictor, which makes them unable to capture disease progression. To fill in this gap, QSP models can be used (Bloomingdale et al., 2022), as they allow to estimate the temporal evolution of AD biomarkers (e.g., Aβ42, sAPP, tau) (Clausznitzer et al., 2018; Geerts et al., 2018; Rollo et al., 2023) and clinical endpoints (e.g., integrated Alzheimer’s Disease Rating Scale, iADRS) (Gueorguieva et al., 2023). Therefore, the combined QSP-ML/AI modelling approach may open new opportunities to diagnose and study cognitive function in AD patients.

#### Clinical management

AI-powered devices can offer support and companionship to patients (e.g., chatbots), as well as help caregivers track their patients and identify potential problems (e.g., monitoring systems). Physicians are often required to make treatment decisions based on limited objective information (e.g., biopsy or imaging) about individual patient’s disease status. A computational digital twin platform integrating a mechanistic model of disease with a responder classifier to predict temporal changes in the organ of interest (Venkatapurapu et al., 2022) could help treatment guidance, by providing valuable information (e.g., temporal) about disease progression, drug efficacy and potential adverse effects.

## *pAIns* of combined QSP-ML/AI approaches

As showcased above, there are several opportunities to combine QSP and ML/AI to address meaningful questions in quantitative drug development. However, to guarantee a successful and long-lasting symbiosis, it is important to highlight some of the associated risks and, where possible, how to mitigate them.

### Right Problem, Right Tool

Choosing the right combined ML/AI and QSP approach requires careful consideration of the specific problem being addressed. Moreover, combined methods are not always a guaranteed improvement over using either ML/AI or QSP alone, or even simpler techniques. Studies have shown instances where predictions by ML alone were no better than basic linear regression (Christodoulou et al., 2019), and where simple ODE models provide reasonable insight and predictive ability compared to QSP models (Stein and Looby, 2018).

### Defining Workflows

While both ML/AI and QSP share some tools and terminology, successfully combining them requires more than just common ground. Specialised workflows that choose the best software and programming languages for this integration need to be developed. Furthermore, the underlying assumptions of each component/algorithm should be communicated and understood by all. Poor communication can make it difficult to spot technical problems and hidden biases that can emerge when combining these workflows (McComb et al., 2022). To ensure that results are reproducible, the developed workflows should be transparent and explainable (Shelmerdine et al., 2021).

### Error propagation

Error propagation concerns the transfer of uncertainty and artifacts between QSP and ML/AI. QSP models can be developed with the knowledge of complex biological processes to “fill in the blanks” from limited data, and they can generate valuable data that can be fed into ML algorithms (Bogatu et al., 2023). However, QSP models themselves contain uncertainties, especially in parameter estimates. Uncertainty assessment in QSP models is an area of active research (Ribba et al., 2017), and it is yet unclear how QSP-based uncertainty can be measured and ultimately mitigated when QSP model outputs are subsequently incorporated into ML approaches.

Future research may include (i) quantification of the uncertainty “chain reaction” by developing methods to track and understand how uncertainty propagates from QSP parameters to the final ML outputs and (ii) mitigation of the propagation impact by designing strategies to reduce or manage the uncertainty within the QSP-ML pipeline, potentially by improving parameter estimation or incorporating uncertainty information into the ML training itself (Fan et al., 2023). An additional instance where error propagation may occur is when ML-derived measurements from 2D images are used in 3D QSP models.

### Data leakage

Data leakage during ML/AI modelling occurs when training datasets contain additional information about the system under study, often leading to an inflated performance on training data and, consequently, to an overestimation of the model’s performance accuracy when applied to other data. Such an ML/AI model may generate incidental and artefactual correlations irrelevant to the underlying mechanisms (Yeo and Selvarajoo, 2022). The source of these circumstantial associations can be technical (Kaufman et al., 2011), due to data pre-processing (Bouke and Abdullah, 2023), or inherent noise. Therefore, before integrating any results into QSP models, careful identification, quantification, and communication of potential sources of error is essential, especially when they cannot be completely mitigated.

### Data bias

Data bias arises when certain subsets are systematically more likely to be selected than others in a sample (Shelmerdine et al., 2021). For example, historically, pre-clinical/clinical data are predominantly from males (Sandberg et al., 2015). QSP predictions stemming from such data will inevitably reproduce such bias, resulting in potential adverse effects and/or loss of efficacy in the underrepresented subpopulation (Zusterzeel et al., 2014; Ganapathi et al., 2022). To avoid such bias, data should be curated to be as diverse and representative as possible. Where this is not possible, models should only be evaluated in scenarios that adequately reflect how they were constructed, and a well-documented description of the datasets used should be provided.

### Appropriate model validation

Technical issues arising from data selection for ML/AI and QSP modelling include the need for effective validation, which is already a central component in both ML/AI and QSP. As such, when applying a symbiotic approach, appropriate validation datasets need to meet both fields’ requirements, which will likely be challenging. For instance, to minimise data leakage in ML/AI, datasets should attempt to counteract the potentially incidental correlations present in the training dataset (Vokinger et al., 2021), whilst any dataset used for QSP model construction should provide complementary perturbations of the system to allow discrimination among models describing distinct mechanisms (Ribba et al., 2017).

### Data collection and curation

Lack of standardised practices for data collection and handling makes meaningful dataset comparisons from different sites/labs difficult. For example, thrombin generation assays used to assess haematological disorders lacked a standardised protocol for sample handling, leading to methodological inconsistencies between testing sites, which complicated (if not invalidated) quantitative comparisons (De Laat Kremers et al., 2020).

Similarly, clinical datasets often suffer from a lack of completeness and consistency (e.g., missing data, inconsistent structure/formatting), which makes their use in QSP models challenging. Data integration from multiple sources and/or data-sharing between different institutions requires extensive and labour-intensive curation before the data can be confidently used: trained and experienced data annotators are needed to meticulously label large complex datasets for ML algorithms, a challenging task that demands precision and expertise in the field (Ohmann et al., 2011).

When handling longitudinal data, a key question to address is how to handle new information. As more accurate measurements or with higher detection levels are acquired, should the models be retrained using a fixed time window of past data? Or should the window be dynamic? Such a choice would have an impact on how much weight is given to potentially inaccurate older data, ultimately affecting the accuracy of the QSP-ML/AI model outputs. Furthermore, due to the long timescale of drug development, data that is regulation-compliant at the time of dataset creation or algorithm training may need to be curated again to fulfil the requirements of a changing regulatory landscape that restricts information access (Lugg-Widger et al., 2018). Therefore, such systematic model and dataset updates necessitate efficient processing pipelines and storage solutions.

## Discussion

While the potential drawbacks (*pAIns*) associated with the symbiotic QSP and ML/AI approach should not be disregarded, the preliminary successes outlined in the *gAIns* section suggest its feasibility and potential. To fully realise its capacity for drug development, QSP modellers and data scientists in the pharmaceutical industry and in academia should work together with drug regulatory agencies. Such interaction should further highlight *gAIns* and make a concerted effort to identify and overcome *pAIns* arising when applying the symbiotic approach, with the ultimate goal of developing useful drugs efficiently and treating patients effectively.

Both the FDA (U.S. Food and Drug Administration) and the European Medicines Agency (European Medicines Agency, 2023) are already implementing risk-based approaches to regulate AI use in drug development. The proposed framework considers the intended purpose of the AI tool and the potential risk it poses to patients. Under such regulatory guidance, AI systems used to identify potential drug targets or to predict the efficacy/safety of a drug candidate may be subject to less stringent regulations than those directly involved in clinical trial decision-making and drug approval. While the FDA’s regulations for AI in drug development are evolving, the core principles outlined in their guidance echo the points raised in this paper. These principles emphasise the importance of (i) utilising high-quality data for training AI systems; (ii) employing transparent and explainable AI algorithms; (iii) monitoring AI system performance in real-world settings; and (iv) guaranteeing the protection of patient data used in the process.

In summary, the presented examples showcase the vast potential of combined QSP and ML/AI approaches in tackling relevant problems in drug discovery and drug development. We have also emphasised the importance of proactively identifying and mitigating potential risks (*pAIns*) associated with this integration. The clear benefits (*gAIns*) achieved through this symbiosis highlight the need to update the adage “no pain, no gain” to a more nuanced perspective: “some *pAIn*, greater *gAIn*.” This shift reflects the understanding that while challenges exist, the rewards of this combined approach far outweigh them, paving the way for significant advancements in drug discovery, efficient drug development, and improved patient outcomes.

## Data Availability

The original contributions presented in the study are included in the article/Supplementary Material, further inquiries can be directed to the corresponding author.

## References

[B1] Alexandrov T. Saez-Rodriguez J. Saka S. K. (2023). Enablers and challenges of spatial omics, a melting pot of technologies. Mol. Syst. Biol. 19, e10571. Epub ahead of print. PMID: 37842805. 10.15252/msb.202110571 37842805 PMC10632737

[B2] Azer K. Kaddi C. D. Barrett J. S. Bai J. P. F. McQuade S. T. Merrill N. J. (2021). History and future perspectives on the discipline of quantitative systems pharmacology modeling and its applications. Front. Physiol. 12, 637999. PMID: 33841175; PMCID: PMC8027332. 10.3389/fphys.2021.637999 33841175 PMC8027332

[B3] Bergen V. Soldatov R. A. Kharchenko P. V. Theis F. J. (2021). RNA velocity-current challenges and future perspectives. Mol. Syst. Biol. 17 (8), e10282. PMID: 34435732; PMCID: PMC8388041. 10.15252/msb.202110282 34435732 PMC8388041

[B4] Bloomingdale P. Karelina T. Ramakrishnan V. Bakshi S. Véronneau-Veilleux F. Moye M. (2022). Hallmarks of neurodegenerative disease: a systems pharmacology perspective. CPT Pharmacometrics Syst. Pharmacol. 11, 1399–1429. 10.1002/psp4.12852 35894182 PMC9662204

[B5] Bogatu A. Wysocka M. Wysocki O. Butterworth H. Pillai M. Allison J. (2023). Meta-analysis informed machine learning: supporting cytokine storm detection during CAR-T cell Therapy. J. Biomed. Inf. 142, 104367. 10.1016/j.jbi.2023.104367 37105509

[B6] Bouke M. A. Abdullah A. (2023). An empirical study of pattern leakage impact during data preprocessing on machine learning-based intrusion detection models reliability. Expert Syst. Appl. 230 (C), 120715. 10.1016/j.eswa.2023.120715

[B7] Chen X. Kamperschroer C. Wong G. Xuan D. (2019). A modeling framework to characterize cytokine release upon T-cell-engaging bispecific antibody treatment: methodology and opportunities. Clin. Transl. Sci. 12 (6), 600–608. 10.1111/cts.12662 31268236 PMC6853151

[B8] Christodoulou E. Ma J. Collins G. Steyerberg E. Verbakel J. Van Calster B. (2019). A systematic review shows no performance benefit of machine learning over logistic regression for clinical prediction models. J. Clin. Epidemiol. 110, 12–22. 10.1016/j.jclinepi.2019.02.004 30763612

[B9] Clausznitzer D. Pichardo‐Almarza C. Relo A. L. Van Bergeijk J. Van Der Kam E. Laplanche L. (2018). Quantitative systems pharmacology model for alzheimer disease indicates targeting sphingolipid dysregulation as potential treatment option. CPT Pharmacom Syst Pharma 7, 759–770. 10.1002/psp4.12351 PMC6263662 30207429

[B10] De Laat Kremers R. M. W. Ninivaggi M. Devreese K. M. J. de Laat B. (2020). Towards standardization of thrombin generation assays: inventory of thrombin generation methods based on results of an International Society of Thrombosis and Haemostasis Scientific Standardization Committee survey. J. Thrombosis Haemostasis 18 (8), 1893–1899. 10.1111/jth.14863 32319140

[B11] Dugourd A. Kuppe C. Sciacovelli M. Gjerga E. Gabor A. Emdal K. B. (2021). Causal integration of multi-omics data with prior knowledge to generate mechanistic hypotheses. Mol. Syst. Biol. 17 (1), e9730. 10.15252/msb.20209730 33502086 PMC7838823

[B12] European Medicines Agency. (2023). Reflection paper on the use of Artificial Intelligence (AI) in the medicinal product lifecycle.

[B13] Fan X. L. Wang P. Li J. Yang N. (2023). Understanding the predication mechanism of deep learning through error propagation among parameters in strong lensing case. Res. Astronomy Astrophysics 23, 125022. 10.1088/1674-4527/ad0498

[B14] Foo K. S. Lehtinen M. L. Leung C. Y. Lian X. Xu J. Keung W. (2018). Human ISL1^+^ ventricular progenitors self-assemble into an *in vivo* functional heart patch and preserve cardiac function post infarction. Mol. Ther. 2018 Jul5 26 (7), 1644–1659. 10.1016/j.ymthe.2018.02.012 PMC6035340 29606507

[B15] Ganapathi S. Palmer J. Alderman J. E. Calvert M. Espinoza C. Gath J. (2022). Tackling bias in AI health datasets through the STANDING Together initiative. Nat. Med. 28, 2232–2233. 10.1038/s41591-022-01987-w 36163296

[B16] Garrido-Rodriguez M. Zirngibl K. Ivanova O. Lobentanzer S. Saez-Rodriguez J. (2022). Integrating knowledge and omics to decipher mechanisms via large-scale models of signaling networks. Mol. Syst. Biol. 18 (7), e11036. PMID: 35880747; PMCID: PMC9316933. 10.15252/msb.202211036 35880747 PMC9316933

[B17] Geerts H. Spiros A. Roberts P. (2018). Impact of amyloid-beta changes on cognitive outcomes in Alzheimer’s disease: analysis of clinical trials using a quantitative systems pharmacology model. Alz Res. Ther. 10, 14. 10.1186/s13195-018-0343-5 PMC5797372 29394903

[B18] Geerts H. Walker M. Rose R. Bergeler S. Van Der Graaf P. H. Schuck E. (2023). A combined physiologically‐based pharmacokinetic and quantitative systems pharmacology model for modeling amyloid aggregation in Alzheimer’s disease. CPT Pharmacom Syst Pharma 12, 444–461. 10.1002/psp4.12912 PMC10088087 36632701

[B19] Gueorguieva I. Chua L. Willis B. A. Sims J. R. Wessels A. M. (2023). Disease progression model using the integrated Alzheimer’s Disease Rating Scale. Alzheimer’s Dementia 19, 2253–2264. 10.1002/alz.12876 36450003

[B20] Hosny A. Parmar C. Quackenbush J. Schwartz L. H. Aerts H. J. W. L. (2018). Artificial intelligence in radiology. Nat. Rev. Cancer 18 (8), 500–510. PMID: 29777175; PMCID: PMC6268174. 10.1038/s41568-018-0016-5 29777175 PMC6268174

[B21] Hosseini I. Gadkar K. Stefanich E. Li C. C. Sun L. L. Chu Y. W. (2020). Mitigating the risk of cytokine release syndrome in a Phase I trial of CD20/CD3 bispecific antibody mosunetuzumab in NHL: impact of translational system modeling. NPJ Syst. Biol. Appl. 6 (1), 28. 10.1038/s41540-020-00145-7 32859946 PMC7455723

[B22] Irons L. Lai M. Pichardo-Almarza C. Kimko H. (2023). Predicting cytokine release syndrome (CRS) severity: from a data-driven approach to semi-mechanistic modeling methods. ACoP14 Poster.

[B23] Jane B. Justin E. Florian J. Madabushi R. Strauss D. Wang Y. (2021). Quantitative systems pharmacology: landscape analysis of regulatory submissions to the US Food and Drug Administration. CPT Pharmacometrics Syst. Pharmacol. 10 (12), 1479–1484. 10.1002/psp4.12709 34734497 PMC8673997

[B24] Kaufman S. Rosset S. Perlich C. (2011). “Leakage in data mining: formulation, detection, and avoidance,” in In Proceedings of the 17th ACM SIGKDD international conference on Knowledge discovery and data mining (KDD '11) (New York, NY, USA: Association for Computing Machinery), 556–563. 10.1145/2020408.2020496

[B25] Li W. V. Li J. J. (2018). Modeling and analysis of RNA-seq data: a review from a statistical perspective. Quant. Biol. 6, 195–209. 10.1007/s40484-018-0144-7 31456901 PMC6711375

[B26] Liebal U. W. Phan A. N. T. Sudhakar M. Raman K. Blank L. M. (2020). Machine learning applications for mass spectrometry-based metabolomics. Metabolites 10 (6), 243. PMID: 32545768; PMCID: PMC7345470. 10.3390/metabo10060243 32545768 PMC7345470

[B27] Lugg-Widger F. V. Angel L. Cannings-John R. Hood K. Hughes K. Moody G. (2018). Challenges in accessing routinely collected data from multiple providers in the UK for primary studies: managing the morass. Int. J. Popul. Data Sci. 3 (3), 432. 10.23889/ijpds.v3i3.432 34095522 PMC8142952

[B28] McComb M. Bies R. Ramanathan M. (2022). Machine learning in pharmacometrics: opportunities and challenges. Br. J. Clin. Pharmacol. 88 (4), 1482–1499. Epub 2021 Mar 17. PMID: 33634893. 10.1111/bcp.14801 33634893

[B29] Mokhtari R. Hamidinekoo A. Sutton D. Lewis A. Angermann B. Gehrmann U. (2023). Interpretable histopathology-based prediction of disease relevant features in Inflammatory Bowel Disease biopsies using weakly-supervised deep learning. Proc. Mach. Learn. Res. 227, 479–495. 10.48550/arXiv.2303.12095

[B30] Myszczynska M. A. Ojamies P. N. Lacoste A. M. B. Neil D. Saffari A. Mead R. (2020). Applications of machine learning to diagnosis and treatment of neurodegenerative diseases. Nat. Rev. Neurol. 16, 440–456. 10.1038/s41582-020-0377-8 32669685

[B31] Neely B. A. Dorfer V. Martens L. Bludau I. Bouwmeester R. Degroeve S. (2023). Toward an integrated machine learning model of a proteomics experiment. J. Proteome Res. 22 (3), 681–696. Epub 2023 Feb 6. PMID: 36744821; PMCID: PMC9990124. 10.1021/acs.jproteome.2c00711 36744821 PMC9990124

[B32] Odusami M. Maskeliūnas R. Damaševičius R. Krilavičius T. (2021). Analysis of features of Alzheimer’s disease: detection of early stage from functional brain changes in magnetic resonance images using a finetuned ResNet18 network. Diagnostics 11, 1071. 10.3390/diagnostics11061071 34200832 PMC8230447

[B33] Ohmann C. Kuchinke W. Canham S. Lauritsen J. Salas N. Schade-Brittinger C. (2011)Standard requirements for GCP-compliant data management in multinational clinical trials. Trials 12, 85. 10.1186/1745-6215-12-85 21426576 PMC3074516

[B34] Poch C. Foo K. S. De Angelis M. T. Jennbacken K. Santamaria G. Bähr A. (2022). Migratory and anti-fibrotic programmes define the regenerative potential of human cardiac progenitors. Nat. Cell Biol. 24, 659–671. 10.1038/s41556-022-00899-8 35550611 PMC9106586

[B35] Procopio A. Cesarelli G. Donisi L. Merola A. Amato F. Cosentino C. (2023). Combined mechanistic modeling and machine-learning approaches in systems biology – a systematic literature review. Comput. Methods Programs Biomed. 240, 107681. 10.1016/j.cmpb.2023.107681 37385142

[B36] Qiu X. Zhang Y. Martin-Rufino J. D. Weng C. Hosseinzadeh S. Yang D. (2022). Mapping transcriptomic vector fields of single cells. Cell 185 (4), 690–711.e45. Epub 2022 Feb 1. PMID: 35108499; PMCID: PMC9332140. 10.1016/j.cell.2021.12.045 35108499 PMC9332140

[B37] Ribba B. Grimm H. P. Agoram B. Davies M. R. Gadkar K. Niederer S. (2017). Methodologies for quantitative systems pharmacology (QSP) models: design and estimation. CPT Pharmacometrics Syst. Pharmacol. 6 (8), 496–498. Epub 2017 Jul 11. PMID: 28585415; PMCID: PMC5572127. 10.1002/psp4.12206 28585415 PMC5572127

[B38] Rollo J. Crawford J. Hardy J. (2023). A dynamical systems approach for multiscale synthesis of Alzheimer’s pathogenesis. Neuron 111, 2126–2139. 10.1016/j.neuron.2023.04.018 37172582

[B39] Sandberg K. Umans J. G. Georgetown Consensus Conference Work Group (2015). Recommendations concerning the new U.S. National Institutes of Health initiative to balance the sex of cells and animals in preclinical research. FASEB J. 29, 1646–1652. 10.1096/fj.14-269548 25713032 PMC6137686

[B40] Shelmerdine S. C. Arthurs O. J. Denniston A. Sebire N. J. (2021). Review of study reporting guidelines for clinical studies using artificial intelligence in healthcare. BMJ Health Care Inf. 28 (1), e100385. PMID: 34426417; PMCID: PMC8383863. 10.1136/bmjhci-2021-100385 PMC8383863 34426417

[B41] Singh G. Ramanathan M. (2023). Repurposing artificial intelligence tools for disease modeling: case study of face recognition deficits in neurodegenerative diseases. Clin Pharma Ther. 114, 862–873. 10.1002/cpt.2987 37394678

[B42] Stein A. M. Looby M. (2018). Benchmarking QSP models against simple models: a path to improved comprehension and predictive performance. CPT Pharmacometrics Syst. Pharmacol. 7 (8), 487–489. Epub 2018 Aug 22. PMID: 29761883; PMCID: PMC6118293. 10.1002/psp4.12311 29761883 PMC6118293

[B43] Timmer L. T. van Rooij E. (2022). Defining the pathways of heart regeneration. Nat. Cell Biol. 10.1038/s41556-022-00914-y 35550613

[B44] Uatay A. Gall L. Irons L. Tewari S. G. Zhu X. S. Gibbs M. (2023). Physiological indirect response model to omics-powered quantitative systems pharmacology model. J. Pharm. Sci. S0022-3549 (23), 11–21. 10.1016/j.xphs.2023.10.032 37898164

[B45] U.S. Food and Drug Administration, Artificial intelligence/machine learning (AI/ML)-Based software as a medical device (SaMD) action plan.

[B46] Venkatapurapu S. P. Iwakiri R. Udagawa E. Patidar N. Qi Z. Takayama R. (2022). A computational platform integrating a mechanistic model of crohn's disease for predicting temporal progression of mucosal damage and healing. Adv. Ther. 39 (7), 3225–3247. 10.1007/s12325-022-02144-y 35581423 PMC9239932

[B47] Verma M. Gall L. Biasetti J. Di Veroli G. Pichardo-Almarza C. Gibbs M. (2023). Quantitative systems modeling approaches towards model-informed drug development Perspective through case studies. Front. Syst. Biol. 2. 10.3389/fsysb.2022.1063308

[B48] Vokinger K. N. Feuerriegel S. Kesselheim A. S. (2021). Mitigating bias in machine learning for medicine. Commun. Med. (Lond). 1, 25. PMID: 34522916; PMCID: PMC7611652. 10.1038/s43856-021-00028-w 34522916 PMC7611652

[B49] Weddell J. (2023). Mechanistically modeling peripheral cytokine dynamics following bispecific dosing in solid tumors. CPT Pharmacometrics Syst. Pharmacol. 12 (11), 1726–1737. 10.1002/psp4.12928 36710368 PMC10681545

[B50] Yeo H. C. Selvarajoo K. (2022). Machine learning alternative to systems biology should not solely depend on data. Brief. Bioinform 23 (6), bbac436. PMID: 36184188; PMCID: PMC9677488. 10.1093/bib/bbac436 36184188 PMC9677488

[B51] Zhang T. Androulakis I. P. Bonate P. Cheng L. Helikar T. Parikh J. (2022). Two heads are better than one: current landscape of integrating QSP and machine learning: an ISoP QSP SIG white paper by the working group on the integration of quantitative systems pharmacology and machine learning. J. Pharmacokinet. Pharmacodyn. 49 (1), 5–18. 10.1007/s10928-022-09805-z 35103884 PMC8837505

[B52] Zusterzeel R. Selzman K. A. Sanders W. E. Caños D. A. O’Callaghan K. M. Carpenter J. L. (2014). Cardiac resynchronization therapy in women: US Food and Drug Administration meta-analysis of patient-level data. JAMA Intern Med. 174 (8), 1340–1348. 10.1001/jamainternmed.2014.2717 25090172

